# Synergism versus Additivity: Defining the Interactions between Common Disinfectants

**DOI:** 10.1128/mBio.02281-21

**Published:** 2021-09-21

**Authors:** Daniel J. Noel, C. William Keevil, Sandra A. Wilks

**Affiliations:** a School of Biological Sciences, University of Southampton, Southampton, United Kingdom; b School of Health Sciences, University of Southampton, Southampton, United Kingdom; CEH-Oxford

**Keywords:** additivity, antimicrobial activity, disinfectants, synergism

## Abstract

Many of the most common disinfectant and sanitizer products are formulations of multiple antimicrobial compounds. Products claiming to contain synergistic formulations are common, although there is often little supporting evidence. The antimicrobial interactions of all pairwise combinations of common disinfectants (benzalkonium chloride, didecyldimethylammonium chloride, polyhexamethylene biguanide, chlorocresol, and bronopol) were classified via checkerboard assay and validated by time-kill analyses. Combinations were tested against Acinetobacter baumannii NCTC 12156, Enterococcus faecalis NCTC 13379, Klebsiella pneumoniae NCTC 13443, and Staphylococcus aureus NCTC 13143. Synergistic interactions were identified only for the combinations of chlorocresol with benzalkonium chloride and chlorocresol with polyhexamethylene biguanide. Synergism was not ubiquitously demonstrated against all species tested and was on the borderline of the synergism threshold. These data demonstrate that synergism between disinfectants is uncommon and circumstantial. Most of the antimicrobial interactions tested were characterized as additive. We suggest that this is due to the broad, nonspecific mechanisms associated with disinfectants not providing an opportunity for the combined activities of these compounds to exceed the sum of their parts.

## INTRODUCTION

Under the U.S. Federal Insecticide, Fungicide, and Rodenticide Act (FIFRA), an antimicrobial pesticide is defined as a biocide that disinfects, sanitizes, or reduces or mitigates growth or development of microorganisms (1). Depending on the application, any antimicrobial pesticides to be sold or distributed in the United States must be registered with either the Environmental Protection Agency (EPA) or Food and Drug Administration (FDA) to ensure that the product meets minimum efficacy and safety standards. Equivalent legislation can be found globally; for example, the European Union enforces the Biocidal Products Regulations (BPR) (2). Inadvertently, the implementation of pesticide regulations has effectively stopped most research into novel antimicrobial compounds due to the cost of development and registration (3). It remains more financially viable for companies to develop formulations containing currently approved active compounds than to risk the cost of developing and attempting to gain authorization for novel antimicrobials. In the EU, this remains the case even after the BPR revision in 2012 which aimed, among other things, to simplify the process of product authorization (2).

As a result, many of the most widely available antimicrobial disinfectant and sanitizer products consist of combinations of a limited number of individual compounds. These products are routinely used as disinfectants and antiseptics in health care settings, in industrial environments, and in day-to-day life in the form of surface sprays, wipes, and hand sanitizers. The central axiom that synergistic interactions can occur between antimicrobials with different mechanisms of action and target sites has resulted in the liberal use of claims of synergy when multicomponent disinfectants products are described and marketed.

However, correctly classifying the type of interaction between antimicrobial agents is a challenging process. For example, inconsistencies regarding the classification of an antimicrobial interaction can arise depending on the method employed (4–7). Common techniques used to investigate antimicrobial interactions include the E-test, time-kill, and checkerboard methods.

Of these methods, the most widely used is the checkerboard assay, a variation of the broth microdilution technique to determine the MIC. In brief, each compound is serially diluted along either the *x* or the *y* axis of a multiwell plate containing growth medium. The wells are then inoculated with the test species and incubated. The output of the test is the fractional inhibitory concentration index (FICI). The lower the FICI value, the higher the level of interaction between the two tested compounds.

The checkerboard method provides a high-throughput technique that can yield a large amount of information about how pairs of antimicrobials interact in a relatively short period of time. Despite this, the checkerboard method does raise significant challenges when it comes to interpreting results and thus classifying antimicrobial interactions. First, the outcome of a checkerboard assay varies depending on the method of interpretation (8), which is especially significant when many published articles do not explicitly state the method of interpretation used. In addition, the FICI thresholds set to define the verdict of an interaction often vary between publications, creating issues around standardization and comparability of results.

Further complications arise when data are compared between species or strains, with publications reporting significant variations in the classification of combined activities of mixtures of both disinfectants (9, 10) and antibiotics (11, 12). This has resulted in the same combinations being reported as synergistic, additive, or indifferent depending on the species or even strain they were tested upon. While certain academic journals have implemented FICI standards when reporting checkerboard data (13–15), these are not universally adhered to between journals, which further contributes to inconsistencies between publications.

A lack of universal consensus on the definitions of antimicrobial interactions raises additional issues. For the purposes of this publication, the definitions used are in accordance with those set out by the European Committee on Antimicrobial Susceptibility Testing (EUCAST). According to EUCAST, an indifferent interaction is one whereby the activity of both components combined is equal to the activity of the most active component (16). An additive interaction has a combined activity no greater than the sum of the activities of each component, while the sum of the individual activities has to be exceeded by the combined activity in order for the interaction to be classed as synergistic (16). Antagonism is the inverse, whereby the activity of both components combined is lower than that of the most active component (16).

These definitions are not universally accepted or adhered to; for example, multiple leading journals in the field do not accept “additive” checkerboard interpretations due to the definition being commonly misunderstood and the intrinsic variability of the method (13, 15). In guidance to authors, it is even suggested that alternative terms, such as “nonsynergistic” (15), be used, thus encouraging researchers to disregard intermediate levels of activity and focus exclusively on interactions that demonstrate synergy (14, 15). This does not mean that these journals completely disregard the existence of additive interactions, but rather that additivity is too difficult to pinpoint and identify reliably using the checkerboard method.

The confusion between additivity and synergism and the methods employed to distinguish between them has led to the two terms often being used interchangeably and potentially erroneously. The confusion is especially significant, as synergism in an antimicrobial formulation is considered “surprising” and thus is patentable (3). This, alongside the increased marketability the “synergy” buzzword brings, provides a commercial incentive to classify such formulations as synergistic, even if the evidence is circumstantial and the definitions are misunderstood. In addition, results that support patentable ideas often remain unpublished in order to prevent potential loss of intellectual property. This means that the evidence required to support a patent is not often subjected to the same scrutiny as peer-reviewed publications.

These factors combined lead to academic and commercial-related research being “all or nothing,” exclusively focusing on synergistic antimicrobial interactions and completely disregarding additive interactions.

With these issues in mind, this study classifies the nature of the interactions between antimicrobials that are commonly used in disinfectant and sanitizer formulations. The compounds examined in this study are listed in Table 1, alongside their mechanisms of action, their applications, and the compounds they are commonly found with in formulations. Previous research has indicated variability between species and strains (9–12); therefore, clinically relevant bacterial species that display a degree of antibiotic resistance were selected in order to provide a stringent test. In addition, strict activity classification thresholds were used to provide clarity and to maintain consistency with the standards set by leading journals in the field (13–15). Additivity was included as a classification due to the context of the test.

**TABLE 1:** Summary of characteristics of the disinfectants used in this study

Compound	Cellular target	Antimicrobial mechanism	Applications	Compounds commonly associated with in formulations
BAC	Membrane	Positively charged quaternary nitrogen interact with anionic lipids, facilitating its own uptake. Adsorption allows hydrophobic tails to insert into the bilayer, causing disruption of lipid organization and breaches in the permeability barrier. Leads to leakage of low-mol-wt material, loss of proton motive force and uncoupling of oxidative phosphorylation (21, 22, 37–39).	Surface disinfection sprays and wipes, eye/ear drops, burn treatments	DDAC, PHMB, ethanol
DDAC	Membrane	Positively charged quaternary nitrogen interact with anionic lipids, facilitating its own uptake. Adsorption allows hydrophobic tails to insert into the bilayer, causing disruption of lipid organization and breaches in the permeability barrier. Leads to leakage of low-mol-wt material, loss of proton motive force and uncoupling of oxidative phosphorylation (21, 37, 39, 40).	Surface disinfection sprays and wipes, sterilization of surgical equipment	BAC, PHMB, ethanol
PHMB	Membrane	Biguanide group interacts and sequesters anionic lipids, forming homogenous lipid domains. This disrupts bilayer organization and leads to permeability of membrane and intracellular leakage (22, 39, 41–43). Evidence also suggests that PHMB is able to translocate across the bacterial membrane, condense bacterial DNA, and prevent DNA replication (44, 45).	Surface disinfection sprays and wipes, wound dressings, contact lens cleaning solution, swimming pool cleaners	BAC, DDAC, ethanol
Bronopol	Proteins; ROS generated target macromolecular structures	Catalyzes oxidation of thiols to disulfides, cross-linking proteins. Changes to protein structure result in impeded functionality. This reaction also produces reactive oxygen species, which damage intracellular structures (46).	Disinfectant, preservative	BAC, DDAC
Chlorocresol	Membrane	Disruption of the permeable barrier and induction of leakage of low-mol-wt intracellular components. Leads to loss of proton motive force and uncoupling of oxidative phosphorylation (21, 47).	Antiseptic, preservative	Ethanol, triclosan

(This work was carried out by Daniel J. Noel as part of his Southampton NIHR Academy Fellowship to fulfill Ph.D. degree training.)

## RESULTS

### MIC.

The MIC results are summarized in Table 2. Benzalkonium chloride (BAC), didecyldimethylammonium chloride (DDAC), polyhexamethylene biguanide (PHMB) and bronopol achieved MICs in the range of 31 μg/ml to 2 μg/ml across all bacterial species tested, while chlorocresol achieved MICs in the considerably higher range of 600 μg/ml to 125 μg/ml. DDAC achieved an MIC that ranged from 8 μg/ml to 2 μg/ml across all tested species. BAC achieved MICs of 8 μg/ml and 5 μg/ml for Enterococcus faecalis and Staphylococcus aureus, respectively, while the MICs for Acinetobacter baumannii and Klebsiella pneumoniae were significantly higher at 31 μg/ml and 20 μg/ml, respectively. Chlorocresol achieved MICs of 200 μg/ml and 125 μg/ml for K. pneumoniae and A. baumannii, respectively, while the MICs for S. aureus and E. faecalis were significantly higher at 600 μg/ml and 500 μg/ml, respectively. Bronopol followed a similar trend, with MICs of 8 μg/ml and 4 μg/ml for K. pneumoniae and A. baumannii, respectively, and 20 μg/ml and 16 μg/ml for S. aureus and E. faecalis, respectively.

**TABLE 2:** MICs of common disinfectants against clinically relevant bacterial species

Bacterial species	MIC (μg/ml)				
	BAC	DDAC	PHMB	Bronopol	Chlorocresol
A. baumannii NCTC 12156	31	8	16	4	125
E. faecalis NCTC 13379	8	4	8	16	500
K. pneumoniae NCTC 13443	20	6	6	8	200
S. aureus NCTC 13143	4	2	6	20	600

The MIC of dimethyl sulfoxide (DMSO) was greater than 100,000 μg/ml (10% [vol/vol]) for all species tested (results not shown). DMSO was therefore not responsible for the activity demonstrated by chlorocresol.

### Checkerboard assay.

**FIG 1:**
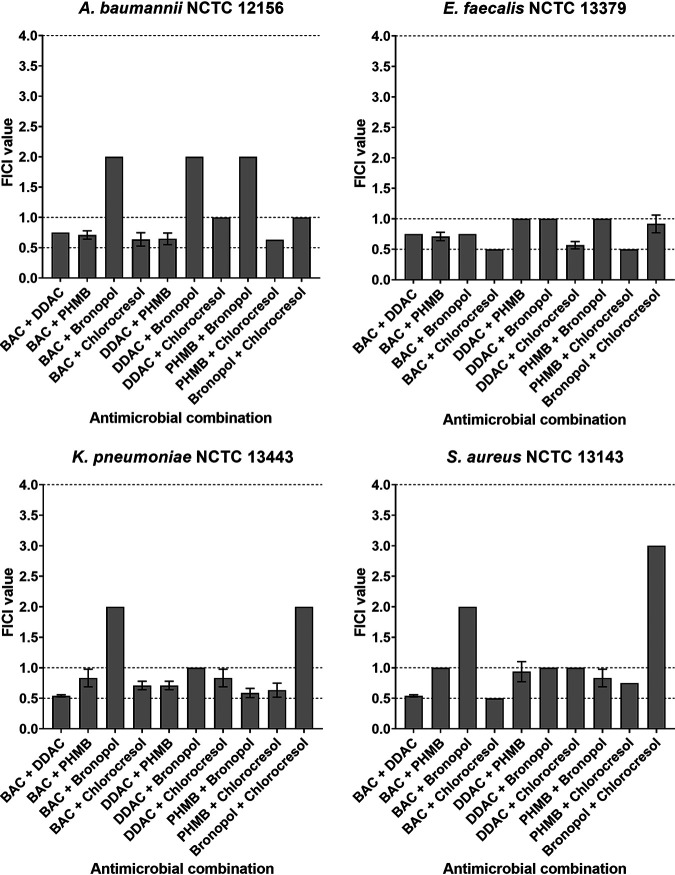
Fractional inhibitory concentration indices (FICIs) of combinations of five common antimicrobial disinfectants. Dotted lines depict the thresholds between synergism (FICI ≤ 0.5), additivity (0.5 < FICI ≤ 1.0), and indifference (1.0 < FICI ≤ 4.0).

The classification of interactions between pairs of disinfectants were evaluated via the checkerboard method. Results are summarized in Table 3 and Fig. 1. The combination of BAC plus chlorocresol demonstrated synergism against S. aureus and E. faecalis, and PHMB plus chlorocresol in combination proved synergistic against E. faecalis. The FICI values of these interactions were all on the threshold of the “synergistic” classification (0.5); therefore, this synergism was considered borderline.

**TABLE 3:** Combined antimicrobial activities of pairwise combinations of five common disinfectants^*a*^

Disinfectant A	Disinfectant B	A. baumannii NCTC 12156			E. faecalis NCTC 13379			K. pneumoniae NCTC 13443			S. aureus NCTC 13143		
		FICI	SD	Activity	FICI	SD	Activity	FICI	SD	Activity	FICI	SD	Activity
BAC	DDAC	0.75	0.00	A	0.75	0.00	A	0.54	0.02	A	0.54	0.02	A
	PHMB	0.71	0.07	A	0.71	0.07	A	0.83	0.14	A	1.00	0.00	A
	Bronopol	2.00	0.00	I	0.75	0.00	A	2.00	0.00	I	2.00	0.00	I
	Chlorocresol	0.64	0.11	A	0.50	0.00	S	0.71	0.07	A	0.50	0.00	S
DDAC	PHMB	0.65	0.10	A	1.00	0.00	A	0.71	0.07	A	0.94	0.17	A
	Bronopol	2.00	0.00	I	1.00	0.00	A	1.00	0.00	A	1.00	0.00	A
	Chlorocresol	1.00	0.00	A	0.57	0.06	A	0.83	0.14	A	1.00	0.00	A
PHMB	Bronopol	2.00	0.00	I	1.00	0.00	A	0.58	0.07	A	0.83	0.14	A
	Chlorocresol	0.63	0.00	A	0.50	0.00	S	0.63	0.12	A	0.75	0.00	A
Bronopol	Chlorocresol	1.00	0.00	A	0.92	0.14	A	2.00	0.00	I	3.00	0.00	I

a Abbreviations: FICI, fractional inhibitory concentration index; SD, standard deviation; BAC, benzalkonium chloride; DDAC, didecyldimethylammonium chloride; PHMB, polyhexamethylene biguanide hydrochloride; A, additive; I, indifferent; S, synergistic.

All combinations of antimicrobials tested demonstrated various degrees of additivity against at least one of the tested species. Most notably, every combination of cationic membrane-active antimicrobials (BAC, DDAC, and PHMB) demonstrated an additive mechanism across all species tested.

Any disinfectant combination that included bronopol produced combined activities that were inconsistent across the different species, with all combinations demonstrating both indifference and additivity that varied in an inconsistent species-dependent manner. Antimicrobial combinations including bronopol were responsible for every indifferent combination tested.

### Time-kill assay.

**FIG 2:**
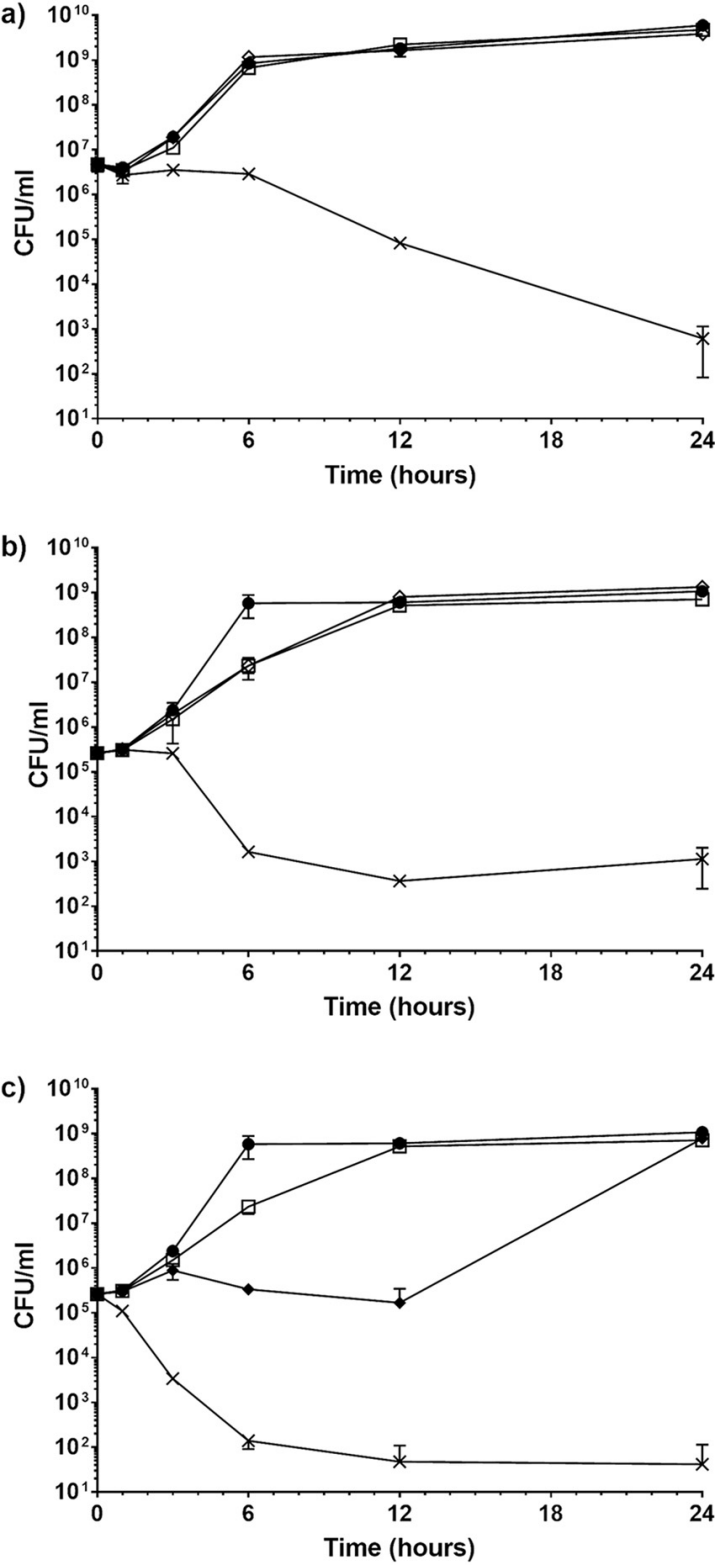
Time-kill curves of synergistic combinations of disinfectants. (a) Staphylococcus aureus NCTC 13143 exposed to a combination of benzalkonium chloride and chlorocresol. •, growth control; ◊, 0.0001% (vol/vol) benzalkonium chloride; □, 0.01% (vol/vol) chlorocresol; ×, 0.0001% (vol/vol) benzalkonium chloride plus 0.01% (vol/vol) chlorocresol. (b) Enterococcus faecalis NCTC 13379 exposed to a combination of benzalkonium chloride and chlorocresol. •, growth control; ◊, 0.0002% (vol/vol) benzalkonium chloride; □, 0.0125% (vol/vol) chlorocresol; ×, 0.0002% (vol/vol) benzalkonium chloride plus 0.0125% (vol/vol) chlorocresol. (c) Enterococcus faecalis NCTC 13379 exposed to a combination of polyhexamethylene biguanide and chlorocresol. •, growth control; ⧫, 0.0002% (vol/vol) polyhexamethylene biguanide; □, 0.0125% (vol/vol) chlorocresol; ×, 0.0002% (vol/vol) polyhexamethylene biguanide plus 0.0125% (vol/vol) chlorocresol. All experiments were performed in triplicate.

Disinfectant combinations previously identified as synergistic were analyzed via the time-kill method. All three combinations demonstrated a ≥2-log_10_ reduction in CFU per milliliter compared to the most active constituent alone and the initial inoculum after 24 h, thus validating the synergistic interactions demonstrated by these three combinations (Fig. 2).

E. faecalis displayed an impaired rate of growth when exposed to the individual disinfectants (Fig. 2b and c), even though they were each below their respective MICs. The cultures demonstrated impeded growth up to 12 h after exposure in comparison to the growth controls (Fig. 2b and c). Despite this, the outcome at the 24-h time point was sufficient for the combined activities of the disinfectants to each demonstrate a synergistic interaction.

## DISCUSSION

Many antimicrobial products used in medical, industrial, and domestic environments consist of formulations of multiple individual disinfectants. Claims are often made regarding the synergistic mechanism of such formulations based on the various compounds present in the solution demonstrating various mechanisms of action.

Despite these claims, there is limited evidence to support synergistic interactions between many of the most common disinfectants. In addition, previous reports indicate that the various methods employed to investigate these interactions can produce inconsistent results (4–7), and various thresholds are often implemented to distinguish between synergistic, additive, or indifferent mechanisms, which can ultimately lead to variation between publications (17–19).

For example, Soudeiha et al. reported an “additive” interaction between colistin and meropenem when tested against A. baumannii clinical isolates *in vitro*, with “additive” FICI values ranging from 0.61 to 1.83 (17). In contrast, when the same antimicrobial combination was tested against A. baumannii clinical isolates by Oliva et al., many of the interactions were reported as “indifferent,” despite the FICI values often being lower than those reported by Soudeiha et al. (17, 18). These discrepancies in the classification of antimicrobial interactions are due to different thresholds being used.

In addition, neither of the studies explicitly defines the terms used (synergy, additivity, or indifference) to classify the antimicrobial interaction between colistin and meropenem (17, 18). Another similar study on the same antimicrobials conducted by Kheshti et al. reported FICI values of between 0.5 and 1 as “partial synergism” (19). The lack of clarification introduces additional ambiguity and hinders the ability to draw an overall conclusion between the published reports (17–19). While these examples investigate antibiotics specifically, the underlying issues extend to all antimicrobial interactions that are examined via the checkerboard method.

Collectively, these factors could lead to the incorrect classification of a combined antimicrobial activity. This is significant, as it may result in consumers placing too much faith in a product, leading to potential inappropriate and ineffective usage. The goal of this study was to investigate the type of interaction between common disinfectants present in formulations and highlight current issues surrounding antimicrobial interaction testing.

The nature of the interactions between 5 common disinfectants when used in pairwise combinations were classified via the widely used checkerboard method. The characteristics of the antimicrobial compounds used in this study are summarized in Table 1. A synergistic interaction between BAC and chlorocresol was observed against E. faecalis and S. aureus and between PHMB and chlorocresol against E. faecalis (Table 3 and Fig. 1). These combinations did not demonstrate synergism against A. baumannii or K. pneumoniae, however, indicating that the synergistic mechanism is species specific.

These synergistic combinations were tested further via the time-kill method. The antimicrobials combined resulted in a ≥5 log reduction in CFU/ml after 24 h compared to when they were used individually, confirming synergistic interactions (Fig. 2).

Indifference was observed in various combinations that contained bronopol, although it is important to note that these indifferent interactions were not consistent across the species tested (Table 3 and Fig. 1). It has been reported that BAC and bronopol synergistically inhibit sulfide production in sulfate-reducing bacteria (20). As antimicrobial activity was measured via sulfide production, it is difficult to draw comparisons between the results. This variation between reports further demonstrates that disinfectant interactions are not ubiquitous and vary in nature depending on the test species and methods used.

Interestingly, it was observed that every combination of cationic membrane-active antimicrobial (BAC, DDAC, and PHMB) interacted additively across all species tested, with FICI values ranging consistently between 0.54 and 1.00. This suggests that disinfectants with similar mechanisms and cellular targets (Table 1) consistently benefit from being in combination, although not to the point of synergism. We propose that this is due to similar-acting compounds having a limited but consistent scope to complement each other’s activities when used in combination. At the sublethal concentrations tested, the cationic membrane-active compounds both disrupt membrane stability and cause intracellular leakage (21–23); thus, they will each mechanistically benefit from the presence of the other. With broadly overlapping mechanisms, the combined activity never has the opportunity to be greater than the sum of its parts; therefore, the interaction is limited to additivity.

Of the 40 test conditions tested, 30 demonstrated an additive interaction against the respective target species (Table 3). In addition, all disinfectant combinations demonstrated at least one additive interaction against the various species. We believe that the abundance of additive antimicrobial interactions is due to the broad, nonselective mechanisms demonstrated by disinfectants. The wide range of cellular targets and high level of activity leaves little room for other additional disinfectant compounds to provide a suitably varying mechanism that would enable a synergistic interaction. As a result, any combined activities would simply be cumulative and would rarely be greater than the sum of the parts. Thus, the majority of the interactions observed are additive. We therefore postulate that finding synergistic combinations of antimicrobials is more challenging when investigating compounds that have nonspecific, broad mechanisms (for example disinfectants) than those that have more specific mechanisms of action (antibiotics).

Despite this observed scarcity of synergistic interactions, claims of synergy are common for disinfectant products. It is possible that products may be being inappropriately classified as synergistic due to the commercial incentives surrounding a “synergistic” claim combined with non-peer-reviewed supporting data and a lack of understanding of the terminology. Clarifying and identifying the differences between any additive and synergistic mechanisms within a disinfectant formulation is of vital importance and should not be dismissed. Synergistic combinations may provide unique and powerful activities that not only influence the effectiveness of the formulation but also impact how it can be effectively used. Overstating the effectiveness of such formulations by erroneously identifying interactions as “synergistic” can lead to consumers placing too much faith in a product, which could lead to inappropriate use.

Furthermore, disinfectants are often under scrutiny by regulatory bodies and can be tightly controlled or withdrawn from use. Regulations vary between countries and regions and are often reviewed and changed; for example, in 2016 and 2017, the FDA banned a total of 24 active ingredients, including triclosan for use in soaps (24, 25). Two of these listed active ingredients applied to specific antimicrobial combinations (25). Other active compounds, including benzalkonium chloride, have had their FDA rulings deferred on a year-by-year basis since 2016 at the request of manufacturers (26–31). This is in order to complete ongoing research into the safety and effectiveness, and as of the time of writing, the most recent deferral will expire on 31 October 2021 (30).

The uncertainty surrounding biocide regulations creates a need for international products to be able to adapt and change their formulations to conform to local regulations. Making a substitution in a formulation is incredibly challenging if the component relies upon a synergistic interaction, as our data suggest that such interactions are very specific and uncommon (Table 3). In contrast, replacing an antimicrobial that provided an additive interaction to a mixture can be achieved relatively easily via a functional analogue, as such interactions are relatively common (Table 3). A formulation that has been inappropriately characterized as synergistic could lead to unnecessary challenge and expense if legislations change and a key component needs replacing. For these reasons, fully understanding the nature of antimicrobial interactions is of paramount importance both to the commercial sector and to consumers.

The observed scarcity of synergistic interactions between broad-spectrum disinfectants also raises the question of whether the benefits of synergistic interactions outweigh the challenges required to identify them. In short, is it worth it? The obvious answer is yes, as there are significant benefits of synergistic interactions. Perhaps most obviously is enhanced antimicrobial activity leading to a higher efficacy, meaning a more effective and reliable product. However, broad-spectrum antimicrobial formulations contain concentrations of active compounds that are typically multiple orders of magnitude greater than the MIC for any likely target organism, and therefore efficacy is not usually a limitation that needs addressing. For example, most supermarket-branded antibacterial sprays and wipes contain between 1,000 and 20,000 ppm BAC, while the MICs against clinically relevant bacterial species lie multiple orders of magnitude lower, in the ranges of 4 to 31 ppm (Table 2). Additionally, there are many widely used disinfectants available that contain only one active component, therefore demonstrating that combined antimicrobial interactions are not necessary for a product to be effective and successful.

A second advantage of synergistic interactions is the ability to minimize resistance development, as targets would have to become resistant to multiple distinct mechanisms simultaneously (11, 32, 33). However, this benefit is not unique to synergistic interactions; it also applies to additive and even indifferent interactions. Furthermore, resistance to disinfectants at optimal concentrations is not a widespread issue that regularly impacts the efficacy of products; thus, it could be argued that this advantage is (currently) a moot point.

An additional advantage of a unique synergistic interaction is that it could enable compounds to be effective against entirely new targets that they otherwise would not work against. However, to our knowledge, there is little evidence to demonstrate this occurring specifically in the case of disinfectant combinations.

With multiple academic journals not accepting additive interactions (13), the current bar is set at distinguishing between synergistic interactions and everything else. However, synergistic interactions do not necessarily provide any discernible advantages over additive interactions with regard to the quality and functionality of a disinfectant product. We therefore question whether these standards are necessary and suggest that the focus instead be shifted to distinguishing between additive and indifferent interactions when the combined activity of broad-spectrum disinfectants is assessed.

It is important to note that disinfectant products routinely contain more than two “active” components, alongside “inactive” additives such as solvents, surfactants, emulsion stabilizers, and fragrance enhancers. Such formulations therefore contain a network of complex interactions between multiple active and inactive compounds, which will inevitably influence the overall product efficacy. To our knowledge, the interactions between common inactive components and active disinfectants within a formulation have not been explored in the literature. Furthermore, the complex interaction networks in combinations of more than two disinfectants have not been characterized. This study comprehensively and systematically classifies the interactions between common disinfectants, representing an important initial step toward fully elucidating the interaction networks that underpin the efficacy of disinfectant formulations used ubiquitously across the world.

### Conclusions.

Disinfectant formulations are globally depended upon in health care environments, in industrial settings, and in day-to-day life. Their use as an infection control measure is critical, especially as the world looks for sustainable routes out of the coronavirus disease 2019 (COVID-19) pandemic. Many common formulations claim to be or are advertised as synergistic. However, the vocabulary surrounding synergism, additivity, and indifference between antimicrobial compounds is often poorly understood and regularly misused. Understanding and not overstating the nature of these interactions is critical because it influences the correct usage of antimicrobial formulations and also dictates the viability of substituting antimicrobials for functional analogues in the event of regulatory changes.

These data demonstrate that synergism between common disinfectants is a rare occurrence and any synergistic mechanisms are not necessarily ubiquitous across bacterial species. The majority of the interactions were characterized as additive, which we suggest is likely due to the broad range of cellular targets providing little opportunity for the activity of any given antimicrobial combination to be greater than the sum of its parts. We therefore question whether the current emphasis on synergistic interactions in academia and product development is necessary in the context of broad-spectrum disinfectants. Synergistic interactions are not likely to provide any discernible or impactful benefit over additive interactions in terms of the quality of the final product.

## MATERIALS AND METHODS

### Bacterial strains and growth media.

The following bacterial strains were used in this study: Acinetobacter baumannii NCTC 12156, Enterococcus faecalis NCTC 13379, Klebsiella pneumoniae NCTC 13443, and Staphylococcus aureus NCTC 13143. The strains were selected due to their clinical relevance and impact on health care-associated infections (34). All bacterial strains were cultured in 10 ml Mueller-Hinton broth (MHB) (Thermo Scientific) overnight at 37°C. Bacterial stocks were standardized to a final test suspension of 5 × 10^5^ CFU/ml.

### Stock solutions of antimicrobial compounds.

Antimicrobial compounds were selected based on their presence in commercial antimicrobial formulations. BAC and DDAC are both quaternary ammonium compounds commonly found as components in antimicrobial formulations. PHMB and phenol derivatives such as chlorocresol are also common components. Bronopol was selected because it acts via a different mechanism in comparison to the other selected compounds. The characteristics of these antimicrobial compounds are summarized in Table 1.

Benzalkonium chloride, didecyldimethylammonium chloride, polyhexamethylene biguanide, and bronopol (all from Thor Specialities Limited) were made up to a stock concentration of 10,000 μg/ml in double-distilled water (ddH_2_O) immediately before testing. Chlorocresol (Lanxess Limited) was made up to a stock concentration of 10,000 μg/ml in undiluted DMSO (Corning) immediately before testing.

### MIC.

The MICs were determined using the broth microdilution method as described by the Clinical and Laboratory Standards Institute (CLSI) (35). Due to the antimicrobial compounds demonstrating a wide range of potential activities, serial dilutions began from 10,000 μg/ml instead of 128 μg/ml as recommended for antibiotics. Experimentation was performed using 96-well plates in triplicate. Plates were incubated at 37°C overnight. The MIC was defined as the lowest concentration of active compound that completely inhibited bacterial growth in the microdilution wells as detected by the unaided eye when the bacterial growth in blank wells was sufficient. The MIC of DMSO was calculated for all tested species to ensure validity of chlorocresol MICs.

### Checkerboard assay.

The checkerboard assay was used to determine the activities of antimicrobial compounds in combination, as described previously (5, 8). Each well of a 96-well plate contained a final volume of 200 μl. Arrangements of antimicrobial compounds were made whereby one compound was serially diluted 2-fold on the horizontal axis and another on the vertical axis, with final concentrations ranging from 4× to 1/128× MIC. Once prepared, each checkerboard plate had 4 sterility controls, 5 growth controls, 10 different concentrations of antimicrobial A alone, 7 different concentrations of antimicrobial B alone, and 70 different combinations of both antimicrobials A and B combined. Checkerboard plates were performed in biologically independent triplicates and were incubated overnight at 37°C. The optical density at 584 nm (OD_584_) of each well was measured using a BMG Labtech FLUOstar Optima microplate reader.

### Analysis.

After normalization, wells that demonstrated an OD_584_ increase of ≥0.1 were considered positive for bacterial growth. Classification of the interaction of any two antimicrobials is based on the fractional inhibitory concentration (FIC): *A*/MIC_A_ = FIC_A_, where *A* is the MIC of compound A when in combination and MIC_A_ is the MIC of compound A when alone.

FICI values were calculated as FIC_A_ + FIC_B_ both from the same well. FICI values were deduced for all nonturbid wells along the turbidity/nonturbidity interface, as described by Bonapace et al. (8). The lowest FICI value was used to characterize the interaction between the two antimicrobial compounds. FICI values were interpreted as synergistic if the FICI was ≤0.5, additive if the FICI was >0.5 and ≤1.0, indifferent if the FICI was >1.0 and ≤4, and antagonistic if the FICI was >4.0. These commonly used thresholds were selected to maintain comparability with other academic publications (13). Thresholds for additivity were included, as nonselective, broad-activity disinfectants were being tested.

### Time-kill assay.

For further validation, disinfectant combinations that were identified as synergistic via the checkerboard method were tested for synergy via time-kill assays as described by CLSI (36). MHB cultures containing 5 × 10^5^ CFU/ml bacteria were exposed to either both antimicrobial compounds, one of the compounds alone, or neither as a growth control. Antimicrobial concentrations were equal to those present in the well exhibiting the lowest FICI value in the checkerboards previously conducted. Cultures had a final volume of 20 ml, with MHB used as the culture medium. Aliquots were taken at 0, 1, 3, 6, 12, and 24 h, and the CFU were quantified via culture analysis. All test conditions were tested in triplicate.

A synergistic interaction was characterized as demonstrating a ≥2 log_10_ reduction in CFU/ml between the combination and its most active constituent alone after 24 h. In addition, the number of CFU per milliliter had to demonstrate a decrease of ≥2 log_10_ below the starting inoculum when the organism was exposed to the antimicrobial combination.
